# Bicuspid Aortic Valve Repair and Single Coronary Ostium

**DOI:** 10.1016/j.jaccas.2024.102656

**Published:** 2024-11-06

**Authors:** Christian Giebels, Peter Fries, Karen B. Abeln, Hans-Joachim Schäfers

**Affiliations:** a Department of Cardiovascular Surgery, Saarland University Medical Center, Homburg/Saar, Germany; b Clinic for Diagnostic and Interventional Radiology, Saarland University Medical Center, Homburg/Saar, Germany; c Department of Cardiac Surgery, University Hospital Quirónsalud, Madrid, Spain; d Saarland University, Saarbrücken, Germany

**Keywords:** aortic regurgitation, aortic valve repair, bicuspid aortic valve, coronary transfer, single coronary ostium

## Abstract

Bicuspid aortic valves may be associated with coronary anomalies. We report a case of a regurgitant bicuspid aortic valve and concomitant single coronary ostium, which we treated by valve repair and coronary transfer.

## Graphical Abstract

**Figure:**
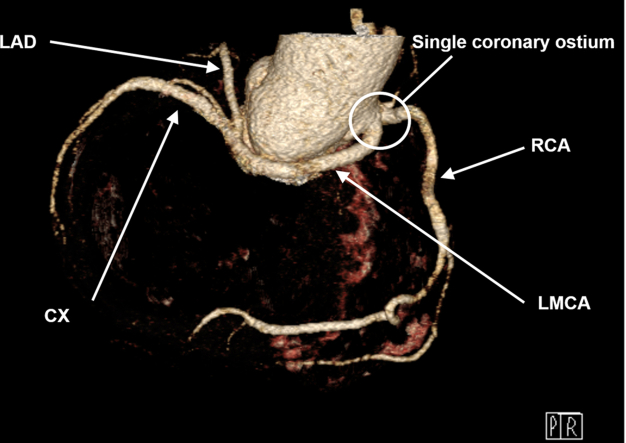

The bicuspid aortic valve (BAV) exists in a spectrum of phenotypes; many affected individuals will require surgery for valve dysfunction or aortic aneurysm.^1^ Of the valve dysfunctions, regurgitation occurs at a relatively young age.^1^ Its standard treatment has been valve replacement, which is associated with a relevant incidence of valve-related morbidity and mortality.^2^ In the past 15 years, repair has become an attractive alternative. In repair, anatomic features of the BAV have to be addressed to achieve good and durable results.^3^ Apart from an annuloplasty, modification of circumferential orientation was found to be beneficial for durability.^4^

**Take-Home Messages**

• The case highlights the importance of imaging before heart valve surgery.
• For successful BAV repair, annular size, cusp configuration, and commissural configuration are important.
• The coronary anatomy has to be taken into consideration when planning the surgical procedure.

Coronary anomalies are relatively frequent in conjunction with BAV.^5^ Most do not require repair, but some—for example, origin of the left main coronary artery (LMCA) from the right sinus—are associated with an increased risk of sudden cardiac death (SCD).^6^ The prognosis of coronary anomalies without interarterial course is less clear, but anatomic features have been associated with an increased risk of SCD or myocardial infarction, including a very long course of the LMCA.^7^

We report a case of a regurgitant BAV with a single coronary ostium, that is, origin of the LMCA from the right sinus with a retroaortic course. To achieve a physiologic result, a decision was made to repair the BAV despite the LMCA anatomy.

## History of Presentation

A 30-year-old asymptomatic man (185 cm; 85 kg) without known previous illnesses had incidentally been diagnosed with regurgitant BAV. His family history was unremarkable, and he was not taking any medications. He was followed and eventually referred for surgery with increasing left ventricular dimensions (left ventricular end-diastolic diameter: 65 mm) and reduced left ventricular function (left ventricular ejection fraction: 48%).

## Investigations

**Figure 1:**
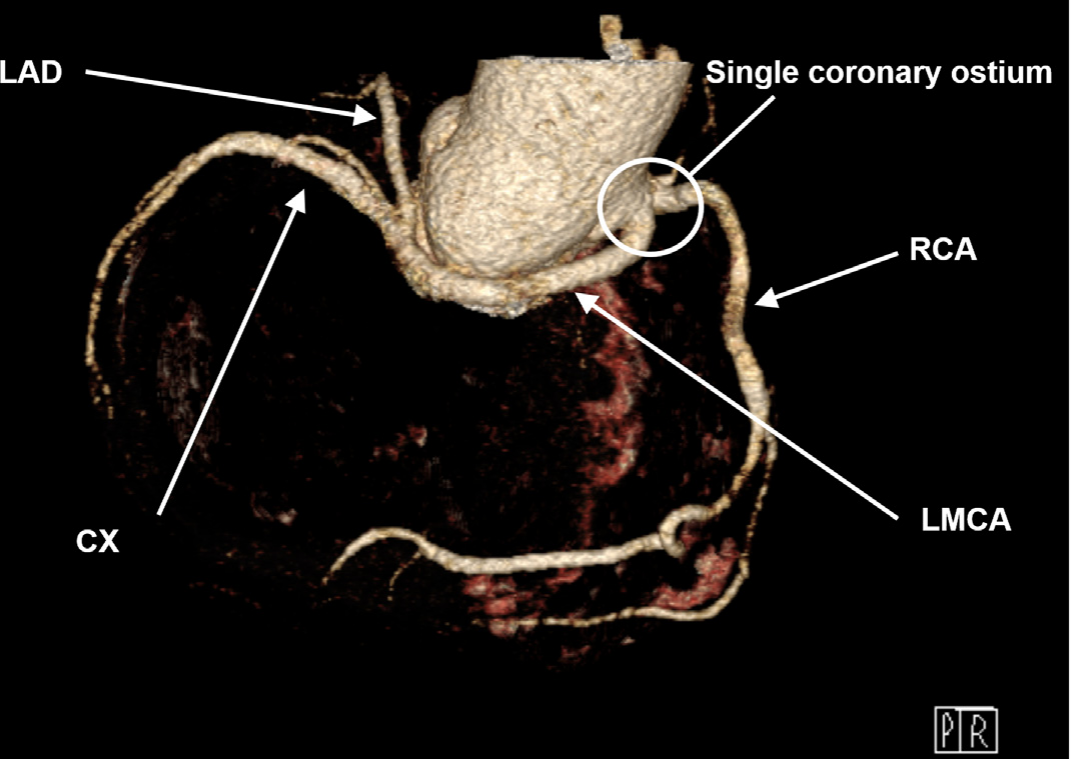
3-Dimensional Reformation of the Coronary Arteries in the Preoperative Computed Tomography Scan Single coronary ostium in the right sinus and long course of the LMCA along the right circumference of the aorta. CX = circumflex artery; LAD = left anterior descending artery; LMCA = left main coronary artery; RCA = right coronary artery.

Preoperative computed tomography revealed a single coronary ostium in the right sinus. The LMCA took a long course along the right circumference of the aorta (Figure 1).

**Figure 2:**
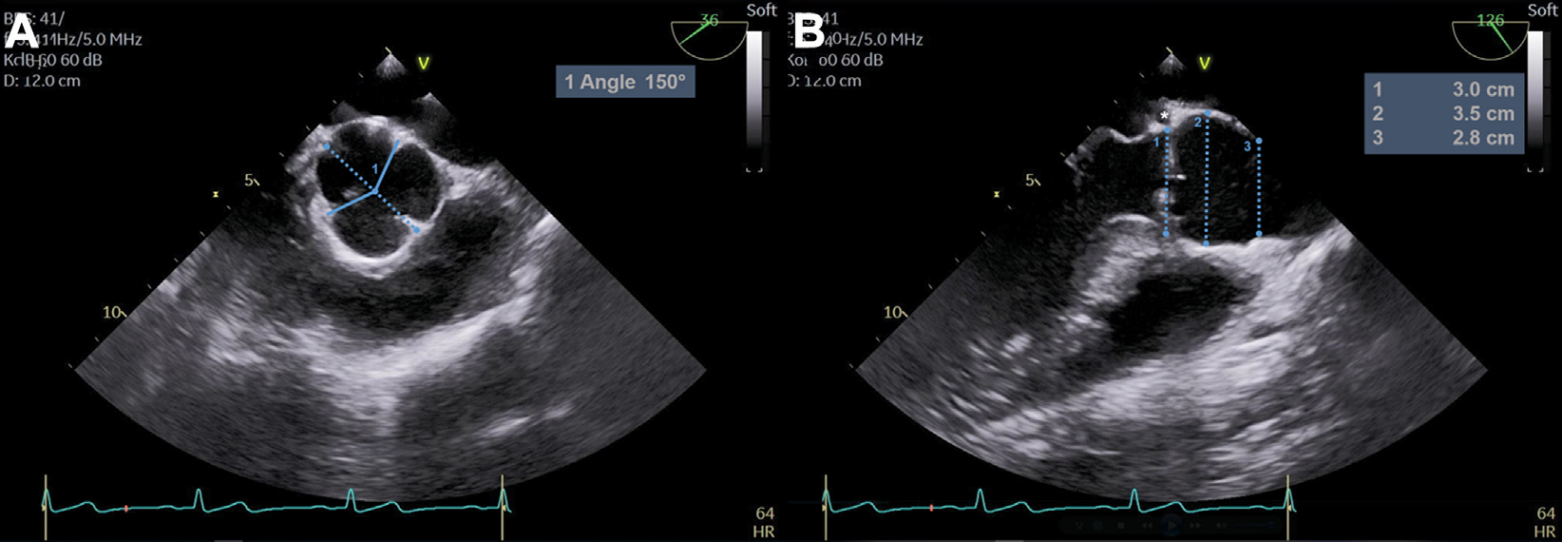
Preoperative Aortic Root Geometry and Dimensions (A) Asymmetric bicuspid aortic valve with fusion of the right and left cusps and a commissural orientation of 150°. (B) The annular diameter is 30 mm. The asterisk shows where the left main coronary artery courses along the aortic base.

The transesophageal echocardiography (TEE) showed an asymmetric BAV with right/left fusion and a commissural orientation of 150° (Figure 2A).^8^ The annular diameter was enlarged (30 mm); other root dimensions were normal (Figure 2B). The LMCA was visible at the lateral base of the aorta (Figure 2B, asterisks). The fused cusp was prolapsing, with severe regurgitation and an eccentric jet toward the anterior mitral leaflet (Videos 1 and 2).

## Management

**Figure 3:**
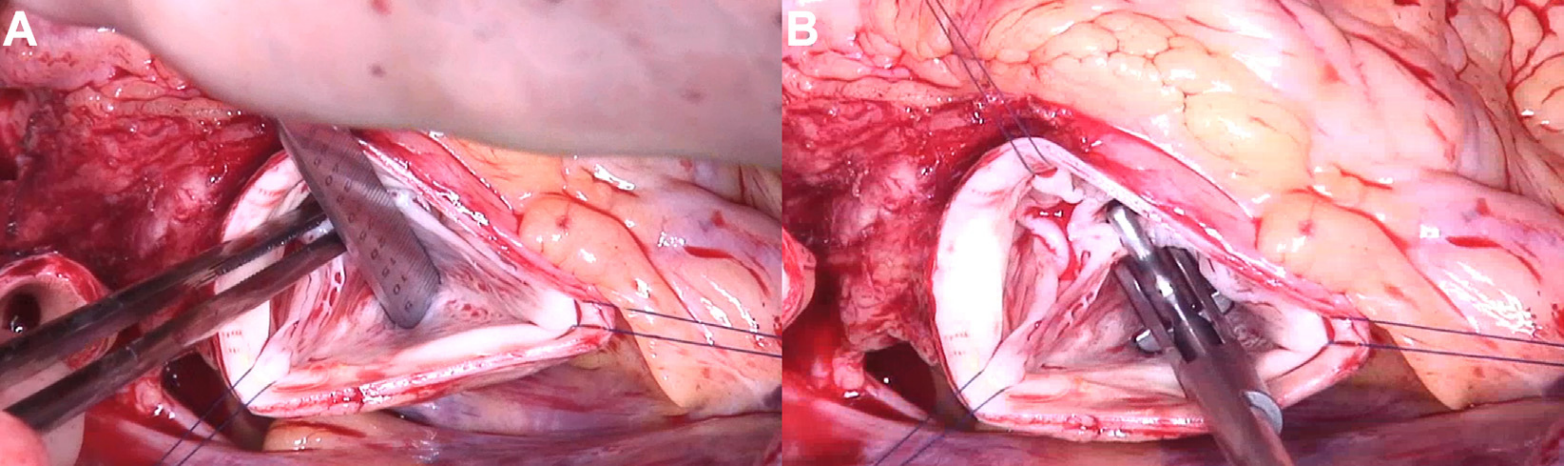
Assessment of the Aortic Valve (A) The geometric height was 23 mm. (B) The effective height was 10 mm.

**Figure 4:**
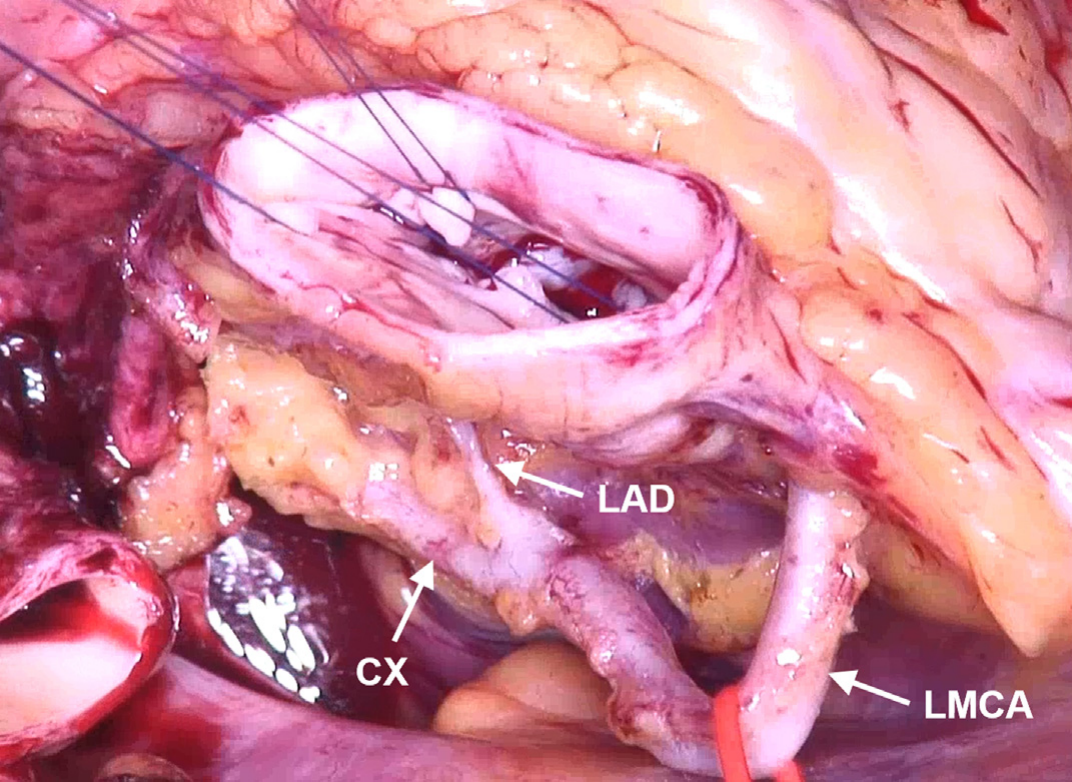
Exposure of the Aortic Root The LMCA courses along the right circumference of the aorta. Abbreviations as in Figure 1.

After median sternotomy and on extracorporeal circulation, the aorta was clamped and transected, and cardioplegia was given. The BAV showed complete fusion between the right and left cusp. The noncoronary cusp had normal size (tissue, ie, geometric height: 23 mm) (Figure 3A)^9^ and configuration (height difference of annulus-free cusp margin, ie, effective height: 10 mm) (Figure 3B).^9^ There was prolapse of the fused cusp, and annular dilatation was confirmed. To prepare for the necessary annuloplasty, the LMCA was exposed. A decision was made to reimplant it into the root with a shorter course; because of the location of its bifurcation (Figure 4), a decision was made to reimplant the LMCA into the noncoronary sinus.

**Figure 5:**
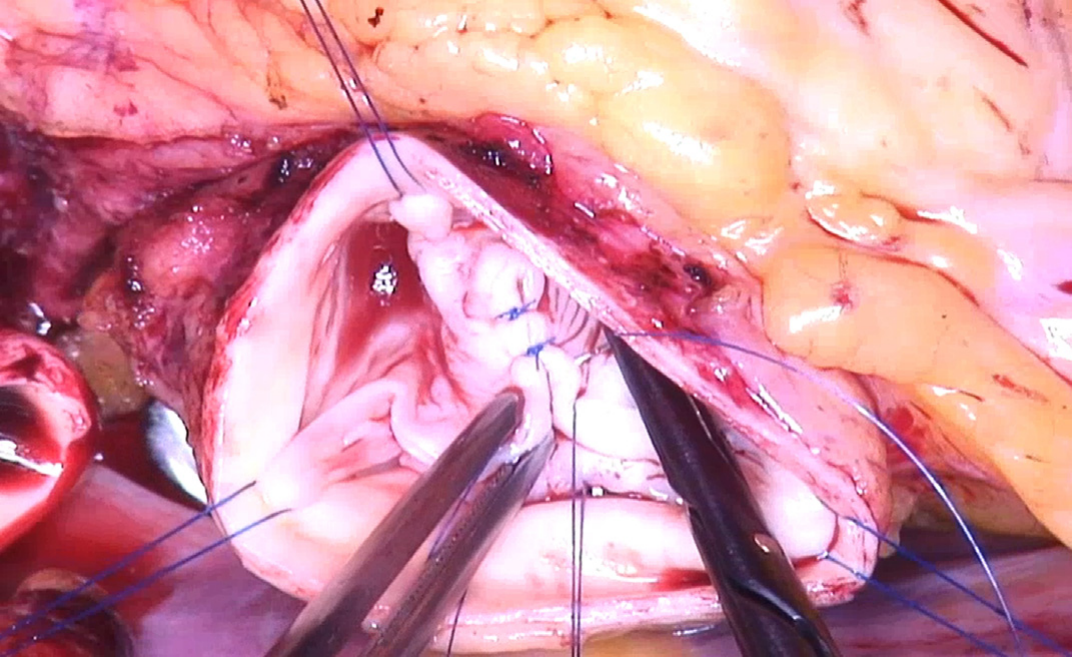
Prolapse Correction The right and left cusps are adapted by central plication until the free margin is at the level of the noncoronary cusp.

**Figure 6:**
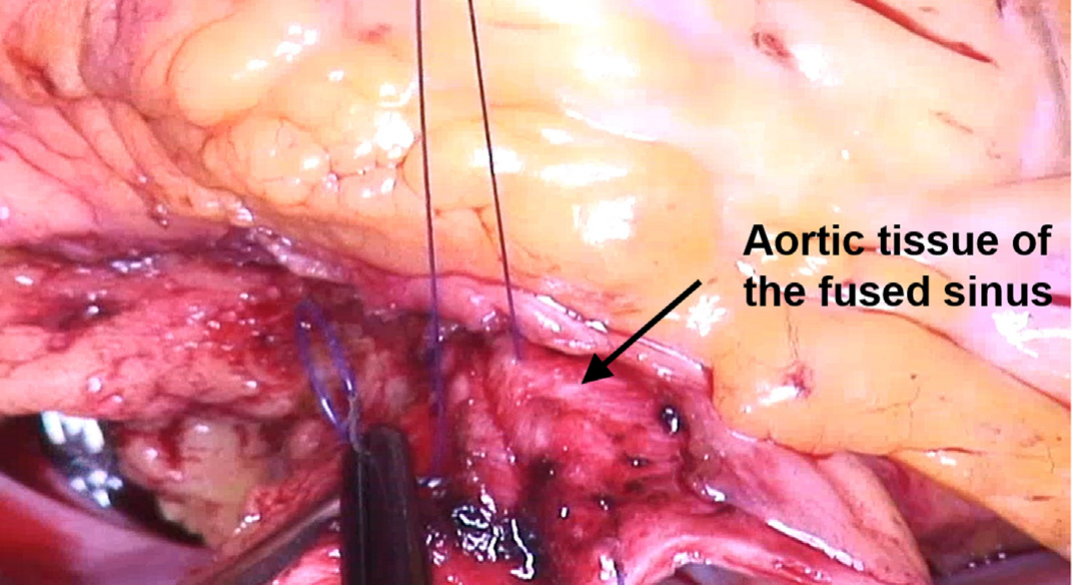
Sinus Plication The circumference of the fused sinus is reduced by a continuous suture.

A suture annuloplasty (Gore-Tex CV-0, WL Gore and Associates) was placed around the basal ring and tied around a 23-mm Hegar dilator.^4^ For prolapse correction, the right and left cusps were adapted by central plication (5-0 polypropylene) until the free margin was at an identical level as the noncoronary cusp (Figure 5). To create a symmetric commissural orientation,^4^ the sinus of the fused cusp was plicated with a continuous suture (Figure 6).

**Figure 7:**
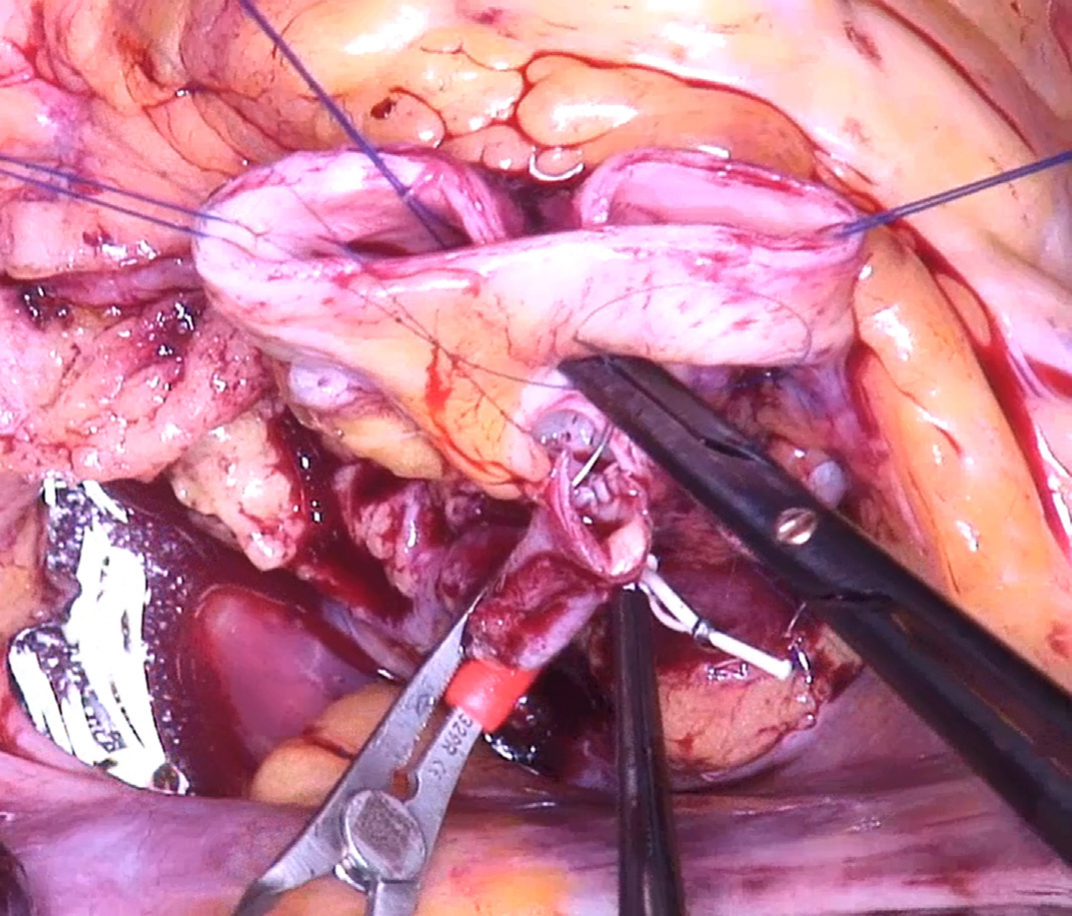
Coronary Transfer The left main coronary artery is implanted into the noncoronary sinus.

**Figure 8:**
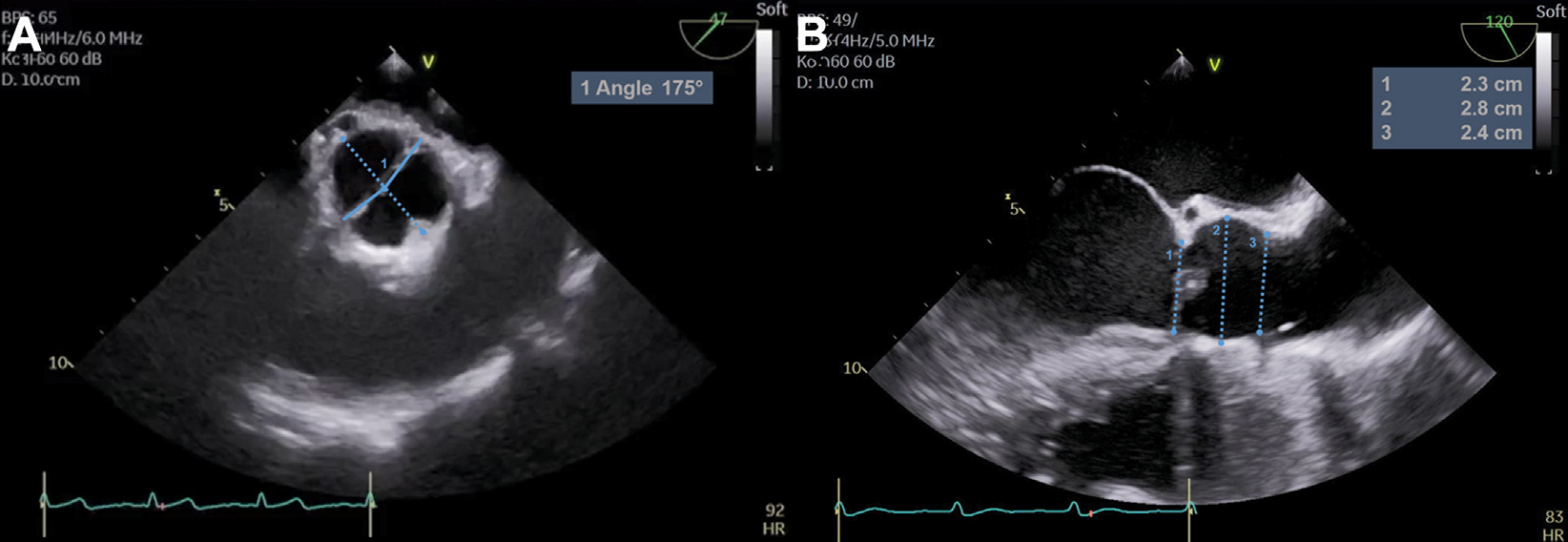
Postoperative Aortic Root Geometry and Dimensions (A) The commissural orientation is nearly symmetric. (B) Annular diameter is normalized.

The LMCA was shortened and implanted into the noncoronary sinus (Figure 7). The aorta was closed, the heart deaired, and the coronary circulation resumed. The heart began to beat in sinus rhythm with a normal QRS complex. The intraoperative TEE showed a competent aortic valve (Video 3) with symmetric commissural orientation and normalized annular diameter (Figure 8).

## Discussion

The experience with BAV repair has shown minimal valve-related complications and good survival if a durable result was obtained.^10^ Specific anatomic features, however, are important for repair durability and systolic valve function.^4^ Apart from avoiding residual cusp prolapse, modification of the annular size and commissural orientation have been predictors for the repair result. The best results have been obtained with an effective height of 9 mm and an annular diameter of ≤25 mm. Symmetric commissural orientation is associated with better durability and lower systolic gradients.^4^

In the present case, the standardized control of cusp dimensions predictably led to the elimination of regurgitation in accordance with our experience. Reducing the circumference of the fused sinuses resulted in a symmetric postoperative configuration. For annular reduction and stabilization, an external annuloplasty is necessary.^4^ For this, the anatomy of the LMCA posed a problem, with possible injury outside the noncoronary sinus, requiring its complete mobilization.

Coronary anomalies are relatively frequent in BAV, and they may create problems during surgery, mainly with administration of cardioplegia. The origin of the LMCA from the right sinus with an interarterial course has been associated with myocardial ischemia and SCD.^6^ The prognostic relevance of the current anomaly is not well known, even though a long LMCA may predispose to spasm, possibly induced by stretching with increased aortic diameters.^7^ We therefore decided to shorten the LMCA by reimplanting it in the noncoronary sinus. Coronary bypass does not appear justified and is associated with vein graft degeneration over time; arterial grafts are prone to early occlusion because of competitive flow. Coronary transfer into a more normal location appears to be the best approach for good early and late results.

## Follow-Up

The patient was discharged on postoperative day 5; 2 years postoperatively, he continues to do well. The last echocardiogram showed normalized left ventricular dimensions (left ventricular end-diastolic diameter: 58 mm) and improved left ventricular function (left ventricular ejection fraction: 61%).

## Conclusions

Complete prolapse repair and normalization of annular dimensions and commissural orientation are important for successful BAV repair even in the presence of coronary anomalies.

## Funding Support and Author Disclosures

The authors have reported that they have no relationships relevant to the contents of this paper to disclose.

## Appendix

Video 1 Preoperative TEE, Long Axis View Prolapse of the fused cusp is clearly visible.

Video 2 Preoperative TEE, Short and Long Axis View Severe regurgitation with an eccentric jet caused by prolapse of the fused cusp.

Video 3 Intraoperative TEE The long-axis view shows a competent aortic valve.
